# Heat shock protein 27 as a predictor of prognosis in patients admitted to hospital with acute COPD exacerbation

**DOI:** 10.1007/s12192-019-01057-0

**Published:** 2019-12-09

**Authors:** Matthias Zimmermann, Denise Traxler, Christine Bekos, Elisabeth Simader, Thomas Mueller, Alexandra Graf, Mitja Lainscak, Robert Marčun, Mitja Košnik, Matjaž Fležar, Aleš Rozman, Peter Korošec, Walter Klepetko, Bernhard Moser, Hendrik J. Ankersmit

**Affiliations:** 1 Christian Doppler Laboratory for Cardiac and Thoracic Diagnosis and Regeneration, Medical University of Vienna, Vienna, Austria; 2 Department of Maxillofacial and Oral Surgery, Medical University of Vienna, Vienna, Austria; 3 Division of Cardiology, Department of Internal Medicine II, Medical University of Vienna, Vienna, Austria; 4 Division of Rheumatology, Department of Internal Medicine III, Medical University of Vienna, Vienna, Austria; 5 Department of Clinical Pathology, Hospital of Bolzano, Bolzano, Italy; 6 Center for Medical Statistics, Informatics and Intelligent Systems, Medical University of Vienna, Vienna, Austria; 7 Division of Cardiology, General Hospital Murska Sobota, Murska Sobota, Slovenia; 8 Faculty of Medicine, University of Ljubljana, Ljubljana, Slovenia; 9 University Clinic of Pulmonary and Allergic Diseases Golnik, Golnik, Slovenia; 10 Division of Thoracic Surgery, Department of Surgery, Medical University of Vienna, Vienna, Austria

**Keywords:** Heat shock protein 27, Chronic obstructive pulmonary disease, Acute exacerbation, Biomarker, Prognosis, Mortality

## Abstract

Episodes of acute exacerbations are major drivers of hospitalisation and death from COPD. To date, there are no objective biomarkers of disease activity or biomarkers to predict patient outcome. In this study, 211 patients hospitalised for an acute exacerbation of COPD have been included. At the time of admission, routine blood tests have been performed including complete blood count, C-reactive protein, cardiac troponin T and NT-proBNP. Heat shock protein 27 (HSP27) serum concentrations were determined at time of admission, discharge and 180 days after discharge by ELISA. We were able to demonstrate significantly increased HSP27 serum concentrations in COPD patients at time of admission to hospital as compared to HSP27 concentrations obtained 180 days after discharge. In univariable Cox regression analyses, a HSP27 serum concentration ≥ 3098 pg/mL determined at admission was a predictor of all-cause mortality at 90 days, 180 days, 1 year and 3 years. In multivariable analyses, an increased HSP27 serum concentration at admission retained its prognostic ability with respect to all-cause mortality for up to 1-year follow-up. However, an increased HSP27 serum concentration at admission was not an independent predictor of long-term all-cause mortality at 3 years. Elevated serum HSP27 concentrations significantly predicted short-term mortality in patients admitted to hospital with acute exacerbation of COPD and could help to improve outcomes by identifying high-risk patients.

## Introduction

Chronic obstructive pulmonary disease (COPD) is a major cause of chronic morbidity and mortality worldwide and its prevalence is still expected to rise (Adeloye et al. 2015; Huang et al. 2001). Although there has been substantial improvement in the understanding of COPD pathogenesis over the last years, existing treatments, such as bronchodilators or anti-inflammatory corticosteroids, have no proven disease modifying effect (Barnes and Adcock 2009).

A blunted regulatory T cell response to tobacco smoking has been identified in COPD patients (Barcelo et al. 2008; Lambers et al. 2009). Increased concentrations of CD28nullCD8+ cells have been shown (Hodge et al. 2011) and lymphocyte senescence seems to be associated with loss of molecular chaperone Hsp90 in CD28nullCD8+ T and NKT-like cells (Hodge et al. 2016). This loss is associated with steroid resistant pro-inflammatory lymphocytes and lung function in COPD (Hacker et al. 2009a).

The aim of current therapy is primarily to improve airflow, reduce dyspnea and prevent exacerbation (Barnes et al. 2003). Episodes of acute exacerbations are the major drivers of hospitalisation and death from COPD. There are no objective biomarkers of disease activity or biomarkers to guide therapeutic choices respectively to risk stratify patients for imminent exacerbations. Finding a reliable marker that identifies patients at risk for future hospitalisations in order to modify and optimise a patient’s current therapy regime may significantly enhance prognosis among this group of patients. Regrettably, at this stage, there are no clinical tools or biomarkers to diagnose COPD exacerbations (Sin et al. 2015).

### Characteristics of an ideal biomarker

A biomarker is a “characteristic that is objectively measured and evaluated as an indicator of normal biological processes, pathogenic processes, or pharmacologic responses to a therapeutic intervention” as defined by the National Institutes of Health Biomarkers Definitions Working Group in 1998 (Biomarkers Definitions Working Group 2001). In a clinical context, these characteristics are used in disease detection and monitoring of health states in individuals or across populations. They can be used to identify individuals with a disease or abnormal condition (diagnostic), as an indicator of disease prognosis (prognostic) or for prediction and monitoring of clinical response to an intervention (predictive). Ideally, a biomarker should be safe and easy to measure, accurate, reproducible across sex and age, modifiable with effective therapy, economical and most importantly should enable clinicians to better manage their patients (Sin et al. 2015; Hollander et al. 2017).

### Heat shock proteins in COPD

Although there has been great effort in COPD biomarker discovery in recent years, clinical translation and implementation have not matched these efforts. Possible candidates that have been thoroughly investigated in COPD patients are heat shock proteins (HSPs). HSPs belong to a highly conserved protein family, which are classified according their molecular weight: some are induced in response to multiple stressful events to protect the cells while others are constitutively expressed. Initially, it was believed that HSPs are only present inside the cells. Interestingly, several groups recently reported the extracellular presence of HSPs (De Maio and Vazquez 2013). Secretion into the extracellular milieu during many pathological conditions suggests additional or novel functions of HSPs in addition to their intracellular properties. Extracellular HSPs are implicated in cell-cell communication, activation of immune cells, and promoting anti-inflammatory and anti-platelet responses (Reddy et al. 2018; De et al. 2000).

The heat shock protein 27, which belongs to the group of small heat shock proteins, has been studied thoroughly in patients with COPD. Increased HSP27 serum concentrations in patients with COPD have been reported when compared with healthy non-smokers and smokers (Hacker et al. 2009b; Unver et al. 2016). Correlation of serum HSP27 concentrations with spirometry analysis and high-resolution computed tomography (HR-CT) revealed that HSP27 is an independent prognosticator of air trapping and emphysema in a study cohort of smokers (Jan Ankersmit et al. 2012). In regard to COPD exacerbations, no data on HSP27 serum concentrations exist.

The aim of this study was to investigate the role of serum HSP27 as a prognostic and predictive marker for all-cause mortality in patients hospitalised due to an episode of acute exacerbation of COPD (AECOPD) up to 3 years. Prognostication of patients with AECOPD could help to improve outcomes by identifying high-risk patients who might potentially benefit from intensive inpatient monitoring and treatment.

We therefore made a post hoc analysis in patients hospitalised due to acute exacerbation, which had been included in a controlled clinical trial to assess the effectiveness of discharge coordinator intervention compared to care as usual in patients with COPD. Retrospective serum HSP27 measurements were performed at time of hospitalisation, respectively, at later stages and correlated with survival data. Analytical performance characteristics have been determined earlier (Zimmermann et al. 2016).

## Materials and methods

### Study subjects

The study protocol has been approved by the National Ethics Committee of the Republic of Slovenia and is registered at ClinicalTrials.gov (NCT01225627). Informed and written consent was obtained from each subject included in the study and all clinical and laboratory tests were performed in accordance with the Declaration of Helsinki and the guidelines for Good Clinical Practice of the Medical University of Vienna. A detailed study protocol and main findings have been published previously (Farkas et al. 2011; Lainscak et al. 2013).

From the prospectively enrolled 253 participants, post hoc analyses were performed with 211 patients due to missing values or blood samples. All of them were admitted for AECOPD between November 2009 and December 2011 at the University Clinic of Pulmonary and Allergic Diseases Golnik, Slovenia. Inclusion and exclusion criteria have been described previously (Farkas et al. 2011). Blood serum samples were collected at admission to hospital, at time of discharge and 180 days after discharge. At the time of admission, routine laboratory parameters were determined including complete blood count, C-reactive protein (CRP), cardiac troponin T (cTnT) and (N-terminal pro-brain natriuretic peptide (NT-proBNP). CRP was measured with an immunoturbidimetric method, cTNT (high sensitivity) and NT-proBNP using an Electro-Chemiluminescence-Immunoassay (ECLIA, Elecsys 2010, Roche Diagnostics). Additionally, serum was obtained after centrifugation of blood samples and aliquots were stored at − 80 °C until further testing.

Endpoint was all-cause mortality. Each patient was followed for 3 years (i.e., exactly 1095 days after admission to hospital) or until death if occurred earlier. Outcome data were available for all patients.

### Quantification of serum HSP27

In a previously published work (Zimmermann et al. 2014), the R&D ELISA DuoSet for total HSP27 (DYC1580, R&D Systems, Minneapolis, MN, USA) showed high diagnostic accuracy in biochemical diagnosis of lung pathologies, when compared to other commercially available ELISA Kits. We therefore used the mentioned ELISA Kit to assess total serum HSP27 concentrations in one batch analysis according to the manufacturer’s protocol.

### Determination of cutoff values

The cutoff values of HSP27 serum concentrations at admission respectively age for Kaplan-Meier curve analysis and Cox proportional hazard models for mortality calculations were determined using Cutoff Finder version 2.1, a freely available R functions-based web application (Budczies et al. 2012), identifying values with highest diagnostic accuracy.

For cardiac biomarkers, the following cutoff values were used for survival analysis: age-adjusted values for NTpro-BNP (ng/L) (Januzzi Jr. et al. 2005) and single values of 14 ng/L for high sensitivity cTnT and 0.5 mg/L for CRP.

### Statistical analysis

Statistical analysis was performed using IBM SPSS Statistics version 23 (SPSS Inc., Chicago, USA) and GraphPad Prism 6 software (GraphPad Software Inc., LA Jolla, CA, USA) was used for data visualisation. HSP27 serum concentrations were compared between different time points using Wilcoxon signed-rank test. Mann-Whitney *U* test and Kruskal-Wallis test were used to compare non-parametric, unpaired variables. Kaplan-Meier curves and log-rank test were used to evaluate time to death for different subgroups (HSP27 high/low, NT-proBNP high/low). Univariable Cox regression models were performed to identify potential influence factors. Multivariable Cox proportional hazard models were then used to further evaluate the prognostic ability of factors being significant in the univariable analysis. Results are expressed as hazard ratio (HR) with corresponding 95% confidence intervals (CI). *P* values were corrected for multiple testing.

Dunn correction was used for post hoc tests in multiple group comparison. All tests were performed in a two-sided manner. Data are presented as median ± interquartile range (ICR). *P* values equal or below 0.05 were considered as statistically significant.

## Results

### Study population

A total of 211 patients were included. Baseline characteristics are summarised in Table 1. They were predominantly males (71%) with advanced COPD (GOLD II-IV) and various comorbidities. All patients received COPD specific therapy according to guidelines. Median age was 72 (IQR, 64–77) years. Follow-up time was 1095 days. The number of deaths at 90 days after discharge was 14 (6.5%), 26 at 180 days (12.0%), 40 after 1 year (18.4%) and 95 after 3 years (43.8%).

**Table 1:** Patient characteristics (*N* = 211). Data are presented as median ± interquartile range (IQR) or number (%). HSP27 data are presented as median ± interquartile range (IQR)

	All patients	HSP27 high ≥ 3098 pg/mL	HSP27 low < 3098 pg/mL	*p* value	Survivors at 3 years	Decedents at 3 years	*p* value
Number	211	51	160		115	96	
Age (median + IQR)	72 (64–77)	76 (72–81)	70 (62–76)	*p* < 0.001	69 (61–76)	74 (69–80)	*p* < 0.001
Gender (male/female)	150 (71%)/61 (29%)	35 (69%)/16 (31%)	115 (72%)/45 (28%)	*p* = 0.723	74 (64%)/41 (36%)	76 (79%)/20 (21%)	*p* = 0.022
GOLD class				*p* = 0.156			*p* = 0.015
GOLD 2	25 (12%)	3 (6%)	22 (14%)		19 (17%)	6 (6%)	
GOLD 3	95 (45%)	28 (55%)	67 (42%)		55 (48%)	40 (42%)	
GOLD 4	91 (43%)	20 (39%)	71 (44%)		41 (36%)	50 (52%)	
LTOT	47 (22%)	10 (20%)	37 (23%)	*p* = 0.701	19 (17%)	28 (29%)	*p* = 0.032
Concomitant diseases							
Heart failure	56 (27%)	19 (37%)	37 (23%)	*p* = 0.067	25 (22%)	31 (32%)	*p* = 0.088
Arterial hypertension	51 (43%)	13 (48%)	38 (42%)	*p* = 0.659	25 (40%)	26 (46%)	*p* = 0.578
Ischemic heart disease	17 (14%)	3 (11%)	14 (15%)	*p* = 0.759	8 (13%)	9 (16%)	*p* = 0.794
Arterial fibrillation	19 (16%)	6 (22%)	13 (14%)	*p* = 0.373	7 (11%)	12 (21%)	*p* = 0.209
Diabetes mellitus	27 (23%)	5 (19%)	22 (24%)	*p* = 0.612	12 (19%)	15 (27%)	*p* = 0.385
BMI (kg/m^2^, median + IQR)	25.7 (23.2–30.4)	23.2 (20.9–28.7)	26.0 (23.7–30.7)	*p* = 0.028	25.4 (23.5–30.1)	26.5 (22.9–30.9)	*p* = 0.946
Tiffenau index (%, median + IQR)	41 (32–52)	45 (35–55)	40 (31–51)	*p* = 0.279	41 (33–51)	41 (30–54)	*p* = 0.804
CRP (mg/L, median + IQR)	23.9 (4.2–75.6)	63.0 (12.7–130.7)	18.4 (3.3–54.4)	*p* = 0.001	18.9 (3.4–74.9)	28.7 (7.2–76.4)	*p* = 0.334
cTnT (ng/L, median + IQR)	0.0 (0.0–18.0)	17.0 (0.0–50.0)	0.0 (0.0–9.5)	*p* < 0.001	0.0 (0.0–11.0)	0.0 (0.0–25.5)	*p* = 0.011
NT-proBNP (ng/L, median + IQR)	471.2 (138.7–1692.0)	1292.0 (432.5–4112.0)	308.6 (117.7–1197.5)	*p* < 0.001	207.9 (89.2–945.1)	1018.5 (324.5–2527.0)	*p* < 0.001
eGFR (CDK-EPI) (ml/min/1.73 m^2^, median + IQR)	81.0 (64.0–92.0)	68.5 (43.0–87.0)	84.0 (70.0–94.0)	*p* < 0.001	81.0 (65.0–92.0)	80.5 (63.0–92.0)	*p* = 0.835
HSP27—admission (pg/ml, median + IQR)	2227 (1560–3070)	3615 (3296–4344)	1931 (1425–2384.9)	*p* < 0.001	2205 (1481–2802)	2257 (1713–3217)	*p* = 0.225
HSP27—discharge (pg/ml, median + IQR)	2037 (1504–2836)	2783 (2269–3678)	1857 (1419–2521)	*p* < 0.001	2002 (1496–2836)	2156 (1510–2783)	*p* = 0.961
HSP27—180 days (pg/ml, median + IQR)	1636 (1333–2405)	2169 (1636–3605)	1580 (1305–2130)	*p* = 0.013	1643 (1355–2418)	1621 (1332–1972)	*p* = 0.705

For survival analysis, cutoff values with highest diagnostic accuracy at time of admission have been identified using a cutoff finder (HSP27 3098 pg/ml respectively age 72 years). Patients who had HSP27 concentrations above the defined cutoff were significantly older, kidney function was decreased and CRP and NT-proBNP concentrations were increased (Table 1).

### HSP27 in COPD patients

Systemic HSP27 concentrations in healthy control patients were assessed in a previous study. Median HSP27 concentration was 1482 pg/ml (IQR 1136–2071 pg/ml) in healthy non-smokers (Zimmermann et al. 2012).

Median HSP27 concentrations in our patient cohort was 2227 (IQR, 1560–3070) pg/ml at admission (211 patients), 2037 (IQR, 1504–2836) pg/ml at discharge (295 patients) and 1636 (IQR, 1333–2405) pg/ml at 180 days after discharge (113 patients). HSP27 concentrations at admission were significantly increased compared to 180 days after discharge (Table 1, *p* = 0.02).

HSP27 concentration at all time points was not significantly associated with GOLD classification. However, HSP27 concentrations correlated significantly with age at admission (*r* = 0.329, *p* < 0.001) and discharge (*r* = 0.244, *p* = 0.001), but not 180 days after discharge (*r* = 0.173, *p* = 0.066).

### Survival analysis

**Fig. 1:**
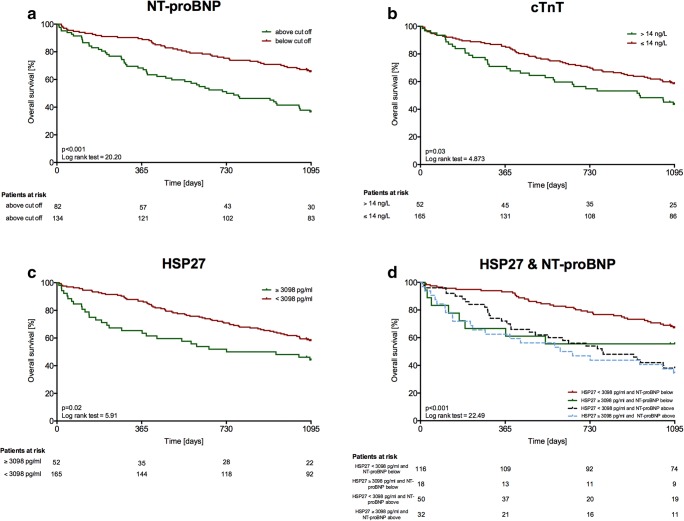
Kaplan-Meier survival stratified by NT-proBNP (**a**), cTnT (**b**), HSP27 (**c**) and combined concentrations of HSP27 and NT-proBNP (**d**) at time of admission

Outcome analysis using Kaplan-Meier survival curves are presented in Fig. 1. Patients with HSP27 concentrations above the cutoff showed a significantly increased risk of death (*p* = 0.02). The median survival time for patients with values above the cutoff was 838 [CI 95% 153–1095] as compared to 1095 [CI 95% 626–1095] days (*p* = 0.007).

Potential risk factors for mortality were selected by univariable analyses. HSP27 demonstrates a significant predictive ability for short- and long-term mortality in the univariable analyses (Table 2, 3 year: HR 1.7, CI 95% 1.1–2.6, *p* = 0.021).

**Table 2:** Univariable Cox proportional hazards analysis for prognostic factor of outcome

Factor	All-cause mortality at 90 days		All-cause mortality at 180 days		All-cause mortality at 1a		All-cause mortality at 3a	
	HR (CI)	*P*	HR (CI)	*P*	HR (CI)	*P*	HR (CI)	*P*
Age (≥ 72a)	1.3 (0.5–3.7)	n.s.	2.2 (1.0–5.1)	n.s.	2.7 (1.3–5.4)	0.003	1.9 (1.3–2.9)	0.001
Gender (male)	0.7 (0.2–2.4)	n.s.	0.9 (0.4–2.1)	n.s.	0.9 (0.5–1.9)	n.s.	0.6 (0.4–1.0)	0.043
GOLD (I, II, III)	2.0 (0.8–4.9)	n.s.	2.3 (1.2–4.7)	0.017	1.8 (1.1–3.0)	0.024	1.6 (1.2–2.2)	0.002
CRP (≥ 0.5 mg/L)	4.5 (0.6–35)	n.s.	2.7 (0.8–9.1)	n.s.	1.6 (0.7–3.6)	n.s.	1.4 (0.9–2.3)	n.s.
cTnT (≥ 14 ng/L)	1.0 (0.3–3.1)	n.s.	1.6 (0.7–3.5)	n.s.	2.1 (1.1–4.0)	0.017	1.6 (1.0–2.4)	0.029
NT-proBNP (age-adjusted)	1.7 (0.6–4.7)	n.s.	2.3 (1.1–5.1)	0.032	3.3 (1.7–6.4)	< 0.001	2.4 (1.6–3.6)	< 0.001
HSP27 (≥ 3098 pg/ml)	4.4 (1.5–12.8)	0.006	4.1 (1.9–8.9)	< 0.001	3.1 (1.7–5.7)	< 0.001	1.7 (1.1–2.6)	0.021

**Fig. 2:**
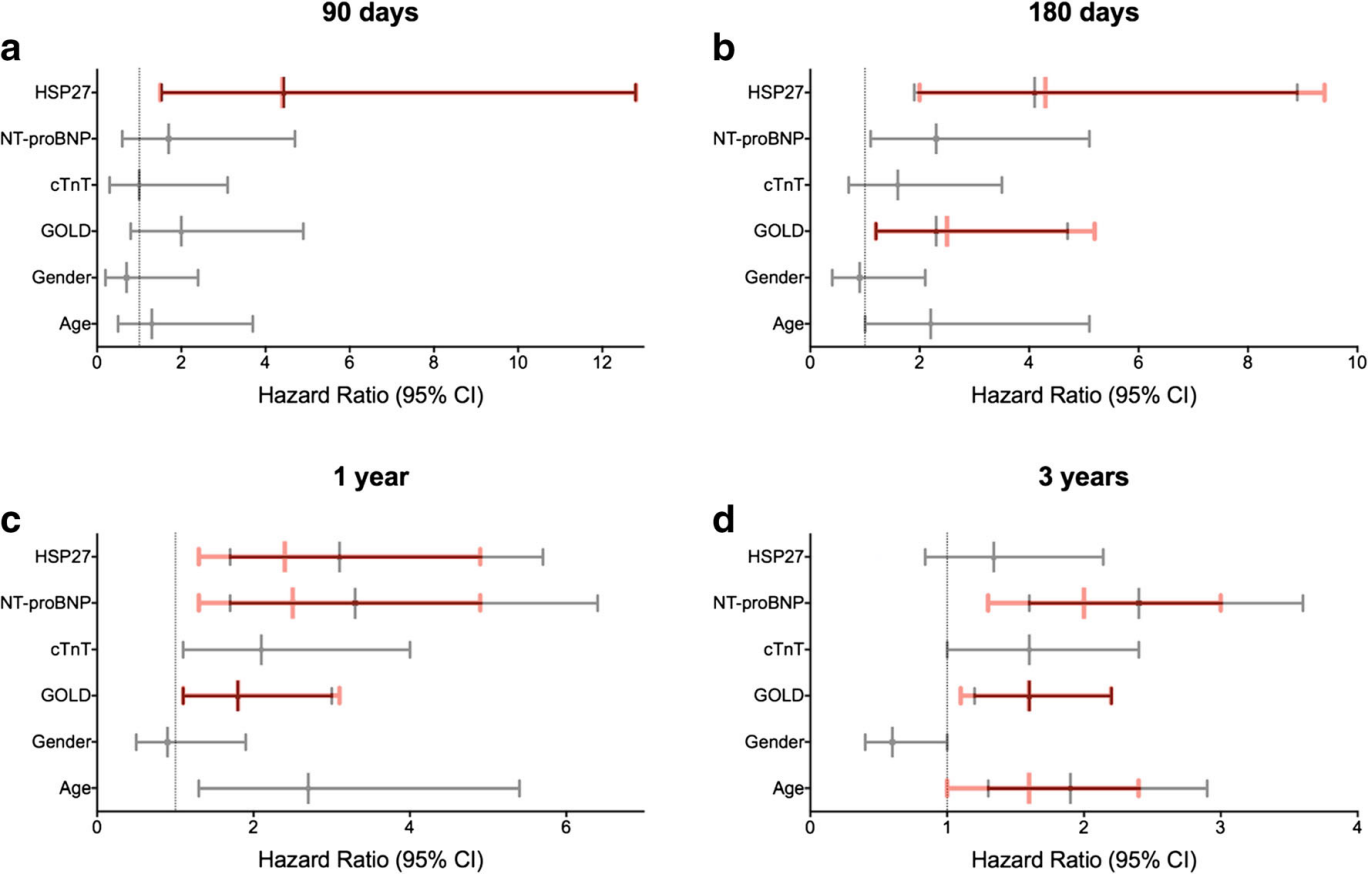
Hazard ratios (HR) and 95% confidence interval (CI) in univariable (grey bar) and multivariable (red bar) Cox regression analysis after 90 days (**a**), 180 days (**b**), 1 year (**c**) and 3 years (**d**). Variables remaining in the model after multivariable analyses are presented in red

Variables that were significant in univariable analysis, such as age, gender, GOLD class, cTnT, and NT-proBNP, were included as adjusting covariates in multivariate analysis to identify potential factors for prediction of mortality. In multivariate Cox proportional hazard regression analysis, age, GOLD class and NT-proBNP were significantly associated with an increased 3 year mortality (Table 3, Fig. 2). However, HSP27 did not remain significant in the model (using backward selection) for 3-year mortality, but it remained significant for short-term outcomes (Table 3, Fig. 2). A significant increased risk of death for patients with higher HSP27 values was found for 90 days (HR, 4.4; 95% CI, 1.5–12.8), 180 days (HR, 4.3; 95% CI, 2.0–9.4) and 1 year (HR, 2.4; 95% CI, 1.3–4.7) after discharge.

**Table 3:** Multivariable Cox proportional hazards analysis with backward selection for prognostic factors of outcome

Factor	90 days		180 days		1a		3a	
	HR (CI)	*P*	HR (CI)	*P*	HR (CI)	*P*	HR (CI)	*P*
Age (≥ 72a)	–	n.s.	–	n.s.	–	n.s.	1.6 (1.0–2.4)	0.047
Gender (male)	–	n.s.	–	n.s.	–	n.s.	–	n.s.
GOLD (I, II, III)	–	n.s.	2.5 (1.2–5.2)	0.013	1.8 (1.1–3.1)	0.031	1.6 (1.1–2.2)	0.008
cTnT (≥ 14 ng/L)	–	n.s.	–	n.s.	–	n.s.	–	n.s.
NT-proBNP (age-adjusted)	–	n.s.	–	n.s.	2.5 (1.3–4.9)	0.009	2.0 (1.3–3.0)	0.001
HSP27 (≥ 3098 pg/ml)	4.4 (1.5–12.8)	0.006	4.3 (2.0–9.4)	< 0.001	2.4 (1.3–4.7)	0.008	–	n.s.

The combination of HSP27 and NT-proBNP in a Kaplan-Meier survival analysis showed a higher survival in the NT-proBNP-low/HSP27-low group when compared with NT-proBNP-low/HSP27-high after 2 years (log-rank test = 5.373, *p* = 0.02).

### Association with other markers

Elevated NT-proBNP and cTnT concentrations at admission were significantly associated with fatal outcome over the follow-up period (Fig. 1). Adjusted Cox proportional hazard models were built using cardiac biomarkers (Table 3). As CRP, cTnT and NT-proBNP have already been identified as markers that predict prognosis in COPD patients after an episode of acute exacerbation (Marcun et al. 2012; Brekke et al. 2008; Hoiseth et al. 2011; Medina et al. 2011; Chang et al. 2011), we have correlated HSP27 concentrations with those markers. At admission, serum concentrations of HSP27 correlated significantly with CRP (*r* = 0.240, *p* < 0.001), cTnT (*r* = 0.353, *p* < 0.001) and NT-proBNP (*r* = 0.429, *p* < 0.001).

Due to its small size, renal elimination of HSP27 is conceivable and we could show a significant association of HSP27 serum concentrations with kidney function. HSP27 correlated with eGFR (*r* = − 0.375, *p* < 0.001).

## Discussion

COPD exacerbations can be life threatening and are the major drivers for hospitalisation of COPD patients. Furthermore, they are believed to contribute to permanent lung changes and permanent decrements in lung function.

In the current study, we were able to demonstrate significantly increased serum HSP27 concentrations in COPD patients at time of admission to hospital due to an episode of acute exacerbation and a decrease of these concentrations after decline of acute symptoms and discharge from hospital. Furthermore, we identified a HSP27 serum cutoff value serving as significant predictor for both short- and long-term mortality using Kaplan-Meier curve analyses and univariate Cox proportional hazard models. We were able to confirm previous findings that NT-proBNP and cTnT predict outcome of these patients.

After adjusting the model for other markers that are known to be associated with mortality in COPD, only HSP27 retained its prognostic performance for short-term mortality.

However, we were not able to significantly correlate serum HSP27 in patients with manifest COPD exacerbation and previous COPD staging according to the GOLD classification (Hacker et al. 2009b).

Although there have been great advances in the understanding of COPD pathogenesis over the last years, biomarker discovery and implementation into the realm of clinics remains challenging. By now, most biomarkers failed to make it beyond the discovery stage. This may be due to the heterogeneity of disease pathogenesis. COPD is not a single entity; airflow limitation is caused by highly variable molecular processes (Lange et al. 2015).

If there is a benefit in serum HSP27 detection in COPD patients still remains unclear. Although HSP27 showed statistical trends to serve as a biomarker in previous works, the diagnostic role remains a matter of debate, as several other conditions and comorbidities may affect serum concentrations. Increased concentrations of circulating HSP27 are reported in different cancers, including lung cancer (Zimmermann et al. 2012), breast cancer (Fanelli et al. 1998; Banerjee et al. 2011), hepatocellular carcinoma (Gruden et al. 2013), pancreatic carcinoma (Liao et al. 2009; Melle et al. 2007), gastric adenocarcinoma (Huang et al. 2010), endometrial cancer and leukaemia. Lastly, elevated concentrations of circulating HSP27 have been observed in patients with cardiovascular disease (Jozefowicz-Okonkwo et al. 2009; Park et al. 2006; Zhang et al. 2017; Jin et al. 2014; Heidari-Bakavoli et al. 2012), multiple sclerosis (Ce et al. 2011), diabetes (Gruden et al. 2008; Jakhotia et al. 2018) or renal injury (Jakhotia et al. 2018; Musial and Zwolinska 2012; Lebherz-Eichinger et al. 2012). All these observations culminate to suggest that HSP27, when found its way to the extracellular milieu, seems to have manifold functions, similar as reported from its intracellular presence. Secreted HSP27 plays an instrumental role in cell-to-cell communication, signalling, immunity and inflammation (Reddy et al. 2017).

Despite lacking disease specificity, current observations suggest that HSP27 serum concentrations can predict disease progress and short-term mortality after COPD exacerbation. Prognostication with HSP27 determination could help to improve outcomes by identifying high-risk patients who might potentially benefit from intensive monitoring and early referral for advanced therapies. It could therefore be a useful tool for clinical decision-making at time of admission.

## Conclusions

Can HSP27 concentrations be used as a predictive and prognostic marker in COPD patients? To obtain a satisfactory answer to this question, further data on a larger population are needed. Results from this initial discovery experiment should be replicated in other cohorts to ensure stability and generalisability of data. More prospective clinical trials are needed to establish optimal cutoff values for survival predictions.

The major limitation of the current study is the fact, that no intervention apart from standard care treatment was performed. So one can only hypothesise that HSP27 serum concentrations can guide therapeutic decisions and enable clinicians to better manage their patients with AECOPD. Furthermore, serum concentrations of HSP27 in healthy individuals may be affected by large intra-individual variation, and assays to date have lacked appropriate standardisation (Zimmermann et al. 2016).

## Abbreviations

AECOPD: Acute exacerbation of chronic obstructive pulmonary disease
CI: Confidence interval
COPD: Chronic obstructive pulmonary disease
CRP: C-reactive protein
ELISA: Enzyme linked immunosorbent assay
GOLD: Global Initiative For Chronic Obstructive Lung Disease
HR: Hazard ratio
HSP: Heat shock protein
IQR: Interquartile range
LTOT: Long-term oxygen therapy
NT-proBNP: N-terminal pro-brain natriuretic protein
cTnT: Cardiac troponin T
